# The Cross-Cultural Competence Inventory: Validity and psychometric properties of the Polish adaptation

**DOI:** 10.1371/journal.pone.0212730

**Published:** 2019-03-07

**Authors:** Krystian Barzykowski, Anna Majda, Paweł Przyłęcki, Małgorzata Szkup

**Affiliations:** 1 Applied Memory Research Laboratory, Institute of Psychology, Jagiellonian University, Kraków, Poland; 2 Faculty of Health Sciences, Institute of Nursing and Midwifery, Jagiellonian University Medical College, Kraków, Poland; 3 Faculty of Health Sciences, Department of Sociology, Medical University of Lodz, Łódź, Poland; 4 Department of Nursing, Pomeranian Medical University in Szczecin, Szczecin, Poland; Middlesex University, UNITED KINGDOM

## Abstract

**Background:**

Due to changes in Polish society resulting from a significant inflow of immigrants to Poland, the need to develop the cultural competences of various professional groups who have contact with immigrants in their work has increased. These groups should include healthcare professionals, especially because of the significant increase in the number of culturally diverse patients. Therefore, medical education in Poland has had to rapidly adapt to this novel situation. For instance, the teaching process should be now more focused on the development and evaluation of the cultural competences of prospective health care workers. However, there is still a lack of standardized, valid and reliable instruments to assess cross-cultural competences among healthcare professionals. The purpose of the present paper was to describe, for the first time, the translation, adaptation, and psychometric evaluation of the Polish version of the Cross-Cultural Competence Inventory.

**Methods:**

Across two studies, we examined psychometric properties of the Cross-Cultural Competence Inventory (CCCI) such as reliability (i.e. internal consistency, test-retest reliability, factor structure) and validity (i.e. theoretical, criteria, convergent). In the first study, 408 participants (75% were healthcare professionals) completed the Polish version of the CCCI and the Positive/Negative Attitude Towards Culturally Divergent People Questionnaire. In the second study, 317 participants (97% were healthcare professionals) completed the CCCI twice, with an interval of at least 22 days. In addition, across two study sessions, participants completed questionnaires constructed to measure (a) cultural intelligence, (b) need for cognitive closure, (c) emphatic sensitiveness, (d) emotional intelligence, (e) self-esteem, (f) social desirability, and (g) personality. Finally, to additionally examine the theoretical validity, 36 professional cross-cultural competence trainers completed the CCCI during a one-session study.

**Results:**

Our findings confirm the reliability and validity of the CCCI. More precisely, in study 1 we proved the theoretical validity and reliability (i.e. internal consistency) of the CCCI. While the assumed structure did not fit the data well, all items were significantly related to the general factor, thus providing strong support for the usage of the total score of the CCCI. In study 2, we additionally estimated the test-retest reliability and theoretical, criterion and convergent validity. Across two studies we were able to successfully confirm these psychometric properties. The reliability was satisfactory and ranged from .83 to .86. We also observed a high and significant positive correlation between CCCI and the Cultural Intelligence Scale, which measures a concept similar to the one measured by CCCI. In addition, a significant relationship between intercultural competences (CCCI) and other variables such as personality, empathic sensitivity, emotional intelligence, self-esteem (positive correlations) and the need for cognitive closure (mainly negative correlation) were demonstrated.

**Conclusions:**

The obtained results support the usage of the CCCI questionnaire in scientific research, such as, for example, among healthcare professionals (nurses, doctors) and students of medical fields (nursing, medicine).

## Introduction

Poland is an EU country that is considered more culturally homogeneous than heterogeneous. According to Eurostat data, the percentage of foreigners in the total population of Poland in 2017 was only 1.7% [1], although in 2011 it was only 0.1% [2]. Thus, Poland is the EU country with the fewest foreigners in relation to the general population. However, one can highlight quite large changes in this area which have been taking place for several years. For instance, recent years have been characterized by a large increase in the number of foreigners coming to Poland. This group includes both economic migrants (mainly from Ukraine), refugees, tourists and other long-term and short-term visitors. The latter groups are mostly overseas students undertaking education at universities in Poland. While in 2005 there were around 10,000 overseas students in Poland, in 2017 that group had increased to over 72,000 [3]. Therefore, it can be reasonably expected that the number of migrants in Poland will rise in the near future.

The changes in recent years in the national structure of Polish society have an impact on various areas of life, including medical care and medical education in Poland. The increase in the number of foreigners living in Poland means that healthcare professionals are dealing more with culturally diverse patients. This situation has led to the necessity of introducing to curriculums content related to intercultural communication. This requirement was introduced in Poland by the government in 2012 [4]. The introduction of these issues in the field of intercultural communication, hitherto rarely undertaken in medical education in Poland, is associated with the necessity of equipping teachers and medical staff with the necessary knowledge, skills and tools to measure cultural competences that can be used in professional work.

While over the years there has been growing interest in cross-cultural competencies, definitions, theoretical models and tools (e.g. [5–24]), to the best of the authors’ knowledge there are no such tools adapted for Polish culture. The existing tools were developed, for example, in the USA, and are perfectly adapted to that specific culture. Tools of this type that examine cultural competences include, for example, *the Nurse Cultural Competence Scale* (NCCS) for nurses who measure cultural awareness, knowledge, sensitivity and cultural skills [14–15], *the Cultural Awareness Scale* (CAS) for nursing students and nurses [10,12], *the Inventory for the Assessment of the Process of Cultural Competency* (IAPCC and IAPCC-R) for health care providers that measure cultural awareness, cultural knowledge, cultural skills and cultural encounters [14,17], *the Cultural Diversity Questionnaire for Nurse Educators* (CDQNE) for nurse teachers [14,18], *the Cultural Knowledge Scale* (CKS) for public health nurses [14,19], and, for example, (as described in Thornson, Thornson & Ross) *the Cross-Cultural Competence Inventory* (CCCI) [20,21], Matsumoto & Hwang’s *Cross-Cultural Sensitivity Scale* (CCSS) and *Intercultural Communication Competence* (ICC) [22], and Bernhard et al.’s *Cross-Cultural Competence of Healthcare Professionals* (CCCHP) [23]. A broad overview of the currently used tools is presented by Loftin et al. [14], Matsumoto & Hwang [22], and Matveen & Merz [24].

Looking at the Polish tools for measuring cultural competences reveals a lack of standardized tools that allow valid, reliable estimation and measurement of cultural competences of medical and health care professionals. In the rare cases when such tools are used, they are used without proper evaluation of psychometric properties such as reliability and validity [25]. In Poland, the number of publications in this area is much smaller, although the interest of researchers in this area is growing. However, Polish authors relatively rarely publish research results on the level of cultural competence [25–27], but rather focus on reviews of foreign literature and the need to develop these competences among medical and health care students, including nurses [28–33].

### The present study

Given the fact that to the best of the authors’ knowledge there are no Polish tools (e.g. questionnaires, scales) allowing measurement of cross-cultural competences, an overriding goal of the present study was to describe, for the first time, the translation, adaptation, and psychometric evaluation of the Polish version of the Cross-Cultural Competence Inventory (henceforth also called CCCI) [20–21].

The CCCI consists of 63 items rated by participants on a 7-point scale. Questions are part of 7 scale dimensions:

1. *Cultural Adaptability*– 18 items relating to, among others, understanding the point of view of people from a different culture and different methods of problem solving, the ability to get used to living in a different culture, communicating with people from different cultures, respect for others' norms, curiosity and willingness to learn about different cultures;
2. *Self-Presentation*– 4 items relating to whether an individual can look straight into the eyes of another person while lying to or cheating him/her, showing a friendly attitude when he/she does not like the person at all;
3. *Tolerance of Uncertainty*– 11 items relating to, among others, whether an individual likes changing plans at the last minute, unpredictable and uncertain situations, disorganized life and speech;
4. *Determination–* 7 items relating to concentration skills, avoiding uncertainty, being decisive;
5. *Engagement–* 11 items: asking inter alia if a person, when feeling stressed, can calm down and think about other things; if an individual likes to talk at a large meeting of friends and acquaintances; if one likes to present him/herself to a group of friends; to what extent one can control his/her own emotions by changing the way he/she thinks about a situation;
6. *Mission Focus*– 7 items relating to whether a person can find several solutions when coping with a problem, understands what is important to others, is effective at work, has the ability to cooperate with others to help them find better ways to accomplish given tasks;
7. *Lie and Social Desirability Scale*–is treated as a control scale evaluating the need to be socially accepted).

The CCCI scale obtained satisfactory psychometric properties in previous studies measured by, for example, internal consistency (Cronbach's α. 70 to .94), test-retest reliability and theoretical, criterion and convergent validity [20].

It can be argued that the CCCI is a comprehensive tool for measuring cultural competencies since it measures three aspects of them: (1) cognitive (culturally specific knowledge, attitude, lack of prejudice, tolerance, flexibility, critical thinking); (2) emotional (cultural empathy, emotional control); and (3) behavioral (experience, initiative, leadership, commitment, communication, effective actions). This is especially true given the fact that the most commonly used definition of cultural competencies refers directly to the three-dimensional model: (1) knowledge–providing culturally specific information; (2) skills–covering multicultural intervention strategies; (3) attitudes–cultural empathy, openness, curiosity, tolerance, flexibility, lack of prejudice in intercultural relations, awareness of one’s own system of values and its limitations, awareness of different perspectives and hierarchy of values, norms and behavioral patterns [34]. Therefore, it can be reasonably argued that the CCCI is an efficient and valid tool for comprehensively measuring cross-cultural competencies. For these reasons, we decided to use the CCCI in the present study.

To adapt the CCCI to Polish culture, we first carefully translated it. Then, we estimated its psychometric properties in two empirical studies. In Study 1, we validated the CCCI’s internal consistency (Cronbach's α). In addition, to address its theoretical validity, we developed The Positive/Negative Attitude Towards Culturally Divergent People Questionnaire. It was expected that people who have not interacted in the past with culturally divergent people are rather reluctant to do so and for this reason should score lower on the CCCI compared to participants who have experience in this area and have a positive attitude towards this group.

In study 2, we provided additional evidence for the test-retest reliability, theoretical validity, criterion validity and convergent validity. Regarding theoretical validity, the CCCI was completed by professional cross-cultural competence trainers. We expected them to score higher on the CCCI compared to the non-professional group of participants. In addition, to examine the criterion validity we developed the Cultural Intelligence Scale [35]. We expected these two tools to be highly positively correlated. Finally, we further investigated the relationship between cultural competencies and other variables that are expected to be correlated with cultural competencies. For instance, it can be argued that factors such as need for cognitive closure, emphatic sensitiveness, emotional intelligence, self-esteem, personality and social desirability may play an important role in the development of cross-cultural competences.

Elaborating further on the relationship between cross-cultural competencies and the aforementioned variables, studies have shown that the need for cognitive closure determines how people think about and experience the lifeworld, and what actions they usually take. Therefore, this trait should be negatively correlated with cultural competences, especially because those with a high need for cognitive closure in social situations prefer order and predictability, are cognitively closed and resistant to change, and experience discomfort when facing ambiguity. On the other hand, a low level of need for cognitive closure requires more tolerance for experiencing uncertainty; it facilitates an open attitude towards incorporating new sets of information (e.g. about a culturally different person), which should be especially helpful during cross-cultural encounters. A low level of need for cognitive closure makes individuals, for example, less inclined to quickly form judgments and assessments, more motivated to perceive others without stereotypies and prejudices, display more flexible behavior (e.g. easily adapting to unexpected situations), and avoid misinterpreting everyday situations [36]. It can be argued that contact with a culturally divergent person requires an open attitude towards ambiguity [37,38]. In addition, emphatic sensitivity may be also an important variable in interpersonal relations and should positively correlate with cross cultural competences. It may be expressed by, for example, the ability to spontaneously adopt a different point of view in everyday life situations; the ability to go beyond one's own ‘self’ when communicating with other people; a tendency to be compassionate towards people; a tendency to experience fear, anxiety, distress, or discomfort in response to other people’s suffering [39]. Importantly, such features are considered useful in intercultural communication [37]. As for emotional intelligence (IE), it should positively correlate with cultural competences. More precisely, IE may be defined as, for example, the ability to [40] regulate mood, to recognize and feel the emotions expressed by other people, to successfully communicate with people characterized by a different style of functioning and emotional expression, or to have and use emotional knowledge (a kind of emotional self-reflection, sensibility in feeling, distinguishing and naming one's emotional states). Emotional intelligence may be especially important for cultural competencies because those who are culturally divergent might have a different style of communicating and expressing emotions [37], which thus requires adequate adaptation. Finally, self-esteem is the attitude towards one’s own self. People with high self-esteem experience positive emotions more often and are more active and persistent, while people with low self-esteem experience more negative emotions, are less active, and are more avoidant of difficulties, challenges and risks [41]. Importantly, given that interacting with a culturally divergent person may be perceived as a challenging situation [38], high self-esteem is expected to be positively correlated with cultural competences. We also expected that personality traits such as extraversion, agreeableness, conscientiousness, emotional stability and intellect [42,43] may also play a positive role in interpersonal and cross-cultural communication and development.

In summary, across the two studies we wanted to thoroughly and carefully examine the reliability and validity of the CCCI. In addition, we wanted to verify the expected relationship between cross-cultural competencies and other factors such as empathy, sensitiveness, need for cognitive closure, emotional intelligence, self-esteem and personality. As argued above, these factors should contribute to the development of cultural competences.

## Study 1

The Jagiellonian Research Ethics Committee approved this study. Written consent for participation was obtained prior to data collection. The privacy of participants was protected as follows: (1) all the information provided by each participant was automatically coded by a number that does not identify any individual; (2) the responses participants provided were collected, coded (turned into numbers) and combined with other participants’ responses (not separately) and because the data were represented as a set of numbers and any identifying information was removed from all non-numerical data, it is impossible for anyone to identify any individual; (3) if an individual chose to stop participating in a study, any data already collected as part of her/his participation was removed from the study records; (4) no participants’ responses will be made public; (5) no-one apart from the authors of the present paper has access to the raw paper questionnaires.

### Participants

A total of 455 individuals participated in the study (315 female, 138 male, 2 participants did not indicate their gender) aged 18–54 (*M* = 21.72, *SD* = 5.80; 3 participants did not indicate their age). All indicated Polish nationality. No incentive was offered for participation in the study. As recommended by the Author of the original scale [20], we excluded 47 participants who scored higher than 15 on the “Lie and Social Desirability” scale. Therefore, the final sample consisted of 408 participants (275 female, 131 male, 2 participants did not indicate their gender) aged 18–54 (*M* = 21.21, *SD* = 4.68). The majority of participants (305, around 75%) were healthcare professionals (e.g. nurse, physiotherapist) and medical or nursing students, while 25% were non-medical students.

### Materials

#### The Cross-Cultural Competence Inventory

The CCCI [20,21] was translated into Polish by two independent translators with high proficiency in English. The translations were then evaluated and adjusted to the final version of the inventory by three of the authors of this paper (K.B., P.P., and M.S.). The final translation was subsequently back-translated into English by an independent translator with high proficiency in English. The back-translated version was then evaluated by the three authors of the present study (K.B., P.P., and M.S.). Any differences between the original and back-translated version of the CCCI were resolved by discussion and the final version of the CCCI was amended accordingly and revised by A.M. The final version of the Cross-Cultural Competence Inventory is provided in the S1 Appendix.

#### The Positive/Negative Attitude Towards Culturally Divergent People Questionnaire

The questionnaire consisted of 10 questions relating to the two main research areas: (1) the participant’s experience in interacting with and attitude towards people from diverse cultural backgrounds, and (2) attitude towards refugees. Regarding the former, participants were asked whether they (a) have ever lived abroad for at least 1 month (Yes/No); (b) have a close relationship with any culturally diverse people (Yes/No); (c) work as healthcare professionals (Yes/No), if Yes; (d) have treated any culturally diverse patients/clients in the past (Yes/No) if Yes; (e) have experienced any troubles or problems during these interactions; (f) would be willing to marry a person from an ethnic minority (e.g. Roma), a different nation (e.g. German), a minority religious community (e.g. Jehovah's Witness). In addition, they decided (Yes/No) whether Europeans, Muslims, Romas and Afro-Americans should be granted the same free health care benefits as Polish citizens. As for their attitude towards refugees, participants were instructed to think about refugees coming to Poland and to answer whether they should be accepted by the Polish government. Importantly, the Study 1 was conducted during the European migrant crisis in 2015, when the European Commission decided to relocate refugees from south European countries to other EU members. This matter triggered a political discussion in Poland and divided the public over the validity of the EC’s decision.

#### Procedure

Participants were tested either individually or in groups. They were informed that they were free to withdraw from the study at any point. The interviewer assured them that their responses would be anonymous, and they could refrain from reporting particularly sensitive information by marking “X” as an answer. Participants first completed the CCCI and then completed the Positive/Negative Attitude Towards Culturally Divergent People Questionnaire.

### Results

#### Descriptive results and reliability: Internal consistency and factorial structure

The overall means for the CCCI are presented in Table 1. As can be seen, the internal consistency of the adapted CCCI inventory (Cronbach's α) was .83 and it ranged from .44 to .83 across the sub-scales.

**Table 1:** Means, standard deviations, Cronbach's α for the CCCI in Study 1 and Study 2.

	Study 1			Study 2									
	Non-Professionals: Group of non-cross-cultural trainers									Correlations: Test-Retest	Professionals: Group of cross-cultural trainers		
				Session 1: Test			Session 2: Retest			CCCI			
	*M*	*SD*	*Cronbach's α*	*M*	*SD*	*Cronbach's α*	*M*	*SD*	*Cronbach's α*	*r*^1^	*M*	*SD*	Statistics^2^^,^^3^
CCCI: Total score	220.40	24.33	.83	218.26	22.12	.83	215.42	22.05	.86	*r*(258) = .79, *p < .001	236.84	21.20	*t(330)* = 4.47, *p* = .001, *q = .019, *d* = .96)
Cultural adaptability	78.32	11.39	.83	79.18	11.31	.86	76.39	11.65	.90	*r*(258) = .79, *p < .001	85.45	10.23	*t(330)* = 2.97, *p* = .003, *q = .025, *d* = .58)
Determination	23.68	5.87	.64	22.53	5.42	.67	22.58	4.96	.67	*r*(258) = .72, *p < .001	28.00	4.68	*t(330)* = 5.40, *p* = .001, *q = .006, *d* = 1.08)
Tolerance	30.34	8.44	.77	29.45	7.80	.77	30.65	8.25	.85	*r*(258) = .77, *p < .001	36.03	7.77	*t(330)* = 4.48, *p* = .001, *q = .013, *d* = .85)
Self-Presentation	13.28	4.47	.65	12.65	4.21	.63	12.98	4.24	.73	*r*(258) = .74, *p < .001	10.55	3.56	*t(330)* = 2.68, *p* = .008, *q = .031, *d* = .54)
Mission focus	31.66	4.23	.54	31.25	4.19	.63	30.20	4.06	.70	*r*(258) = .58, *p < .001	32.35	3.68	*t(330)* = 1.41, *p* = .159, *q* = .038, *d* = .28)
Engagement	43.11	7.19	.70	43.19	6.30	.67	42.61	6.17	.74	*r*(258) = .69, *p < .001	44.45	6.98	*t(330)* = 1.05, *p* = .295, *q* = .050, *d* = .19)
Lie and Social Desirability	9.69	2.75	.44^1^	9.84	2.80	.56^1^	10.07	3.07	.71^1^	*r*(352) = .64, *p < .001	10.55	3.23	*t(330)* = 1.32, *p* = .187, *q* = .044, *d* = .23)

Notes: The average interval between test and re-test in Study 2 was 28.06 ±4.34 days, range = 22 to 47 days.

^1^ Pearson’s correlation coefficient. We calculated Cronbach's α for the Lie and Social Desirability scale without excluding participants who performed highly on this scale.

Significant results are marked with an asterisk (e.g. *q).

^2^ We compared the average results between cross-cultural trainers and non-professional participants’ results obtained during the first session.

^3^ Tests are statistically significant at the corrected *q* = .031 level.

Next, we performed factorial analysis to further examine the factorial structure of the CCCI. In addition we verified the postulated 6-dimensional structure of the CCCI [20,21]. The results indicated that the 6-dimensional structure we postulated was not the best fit to the data: *χ2*(1580) = 4045.82, *p* < .001, *χ2/df* = 2.561, *GFI* = .72, *AGFI* = .70, *NFI* = .48, *RMSEA* = .06, 90% *CI* [.060, .064], *CFI* = .60. All the items were significantly related to the general latent trait (*ps* < .011; standardized regression weights ranging from .20 to .73).

#### Theoretical validity

To analyze the CCCI’s validity, we verified whether participants who demonstrated positive relationships with and/or attitude towards foreign-born populations, minorities and migrants performed higher on the CCCI scale, as might be theoretically expected. For example, we would expect that an individual who has a close and positive relationship with a person from the Roma minority would perform higher on the CCCI compared to someone who has no such experience. To fulfil this goal, we conducted a series of independent t-tests to find differences in the total score of CCCI between participants with positive and negative attitudes that were operationalized as agreeing (positive attitude) or disagreeing (negative attitude) with, for example, allowing immigrants to study at Polish universities. In total, we performed 13 t-tests. To control for multiple comparisons, we chose the False Discovery Rate correction [44]. With *α* = .05, the critical corrected value *q* was .046. The effect size was measured by Cohen's *d* with small, medium, and large effects defined as 0.2, 0.5, and 0.8, respectively [45].

As can be seen in Table 2, participants who declared a positive attitude towards culturally diverse groups of people obtained significantly higher total scores on the CCCI. The only non-significant difference was between people who were for or against providing EU citizens with healthcare benefits within the Polish healthcare system. More precisely, independently of being for or against culturally diverse people, participants were comparable in terms of the average total score in the CCCI.

**Table 2:** Means and standard deviations for CCCI total scores across participants with either a positive or negative attitude towards foreign residents (e.g. refugees, immigrants, foreign-born people) in Study 1.

		Attitude toward foreign residents and minorities				
		Positive: (Yes)		Negative: (No)		
		*M*	*SD*	*M*	*SD*	Statistics
Having a close/friendly relationship with culturally divergent people		230.34	23.49	214.88	22.64	*t(398)* = 6.53, *p* = .001, *q = .012, *d* = .71
Living abroad for at least a month in the past		227.96	23.34	218.41	24.02	*t(405)* = 3.36, *p* = .001, *q = .023, *d* = .38
Would you be willing to marry:	German	223.46	23.88	215.09	24.69	*t(335)* = 2.77, *p* = .006, *q = .031, *d* = .34
	African-American	225.70	23.74	210.78	24.16	*t(320)* = 5.20, *p* = .001, *q = .004, *d* = .64
	Russian	225.43	22.86	213.45	24.81	*t(324)* = 4.27, *p* = .001, *q = .015, *d* = .52
	Roma	232.23	22.50	216.21	23.91	*t(302)* = 4.26, *p* = .001, *q = .019, *d* = .71
	Jehovah's Witness	226.62	24.57	218.57	24.71	*t(313)* = 2.11, *p* = .035, *q = .046, *d* = .33
	Jew	226.41	24.49	211.10	23.10	*t(298)* = 5.19, *p* = .001, *q = .008, *d* = .64
Should refugees from Syria and Iraq be accepted by the Polish government?		228.97	23.60	219.84	24.95	*t(335)* = 2.61, *p* = .009, *q = .035, *d* = .38
Granting free health care to:	Europeans	221.53	24.09	220.65	26.69	*t(362)* = .15, *p* = .884, *q* = .050, *d* = .04
	Muslims	224.71	24.96	216.42	23.84	*t(308)* = 2.91, *p* = .004, *q = .027, *d* = .34
	Romani people	224.28	24.08	216.84	25.25	*t(305)* = 2.53, *p* = .012, *q = .038, *d* = .33
	African-Americans	223.64	24.30	215.82	25.58	*t(306)* = 2.36, *p* = .018, *q = .042, *d* = .33

Note. Tests are statistically significant at the corrected *q* = .046 level (Study 1).

Significant results are marked with an asterisk (e.g. *q).

A positive attitude indicated being open to culturally divergent groups of people (e.g. agreeing to provide them with free of charge medical studies).

In addition, individuals who had no previous practical work experience (e.g. a job or internship) did not differ in the total CCCI score from participants who had already worked as, for example, a nurse (*M* = 221.84, *SD* = 25.02; *M* = 219.75, *SD* = 23.28, respectively; *t*(393) = .85, *p* = .396; *d* = .89). At the same time, experienced participants who had encountered culturally diverse people while working scored significantly higher on the CCCI than individuals who had no similar experience (*M* = 224.09, *SD* = 21.88 vs. *M* = 215.12, *SD* = 23.89, respectively; *t*(187) = 2.62, *p* = .010; *d* = .39). Finally, participants who had had difficulties during such cross-cultural encounters did not differ in the total CCCI score from individuals who had not experienced any difficulties (*M* = 226.31, *SD* = 19.84 vs. *M* = 224.12, *SD* = 23.52, respectively; *t*(82) = .41, *p* = .681; *d* = .10).

### Discussion

#### Reliability and structure of the Cross-Cultural Competence Inventory

The main goal of Study 1 was to analyze the psychometric properties of the Polish version of the Cross-Cultural Competence Inventory. Our findings demonstrated that the CCCI as a measurement tool has good internal consistency. However, while the original version of the CCCI suggested the 6-dimensional factorial structure [20,21], our confirmatory factor analysis (CFA) did not support this expectation. More precisely, the assumed structure did not fit the data well, but all items were significantly related to the general factor. Therefore, while the subscale score analysis should be treated with reservation, our findings provide strong support for the usage of the total score of the CCCI.

#### Validity of the Cross-Cultural Competence Inventory

In order to examine the theoretical validity, we verified whether groups of people differing in terms of their positive vs. negative attitude towards culturally divergent people (e.g. Roma, Jews, Afro-Americans, Syrians and Iraqi refugees) score differently in the CCCI. As might be expected, someone who has high cross-cultural competencies should also be more open to culturally different people compared with someone who has low cross-cultural competencies. Our findings provide strong support for this expectation. First, participants who declared their support for (a) marrying a culturally divergent person, (b) accepting Syrian and Iraqi refugees, (c) granting free healthcare benefits to Roma, African-Americans and Muslims scored higher on the CCCI than individuals who were against these ideas. Importantly, there were no differences between people being for/against granting healthcare benefits to Europeans. This could be expected, since Polish citizens are also Europeans.

Finally, our findings suggest that exposure to cultural diversity may be a crucial factor in developing cross-cultural competences. While participants with or without previous professional experience did not differ in the total CCCI score, those who declared (a) having a close relationship with culturally diverse people, (b) living abroad for at least 1 month, (c) encountering culturally diverse patient/clients during their professional activity scored higher on the CCCI than individuals who had not experienced cultural diversity in the past. Importantly, even facing difficulties and problems during such encounters did not affect participants’ competences. While it may be argued that exposure to cultural diversity enhances cross-cultural competencies, it is also possible that people who are highly culturally competent (i.e. those who are, for example, open to cultural diversity and who have high interpersonal, communication and coping skills) are also more likely to interact with culturally divergent individuals (e.g. not afraid of travelling abroad) in situations in which others may refuse to interact (e.g. while working in a hospital). Importantly, they may also experience such encounters as being generally more positive. Therefore, the exact direction of the relationship between exposure to cultural diversity and cross-cultural competencies is not clear and still needs to be further investigated in future studies.

In summary, all these findings together provide strong support for the theoretical validity of the CCCI.

## Study 2

The aim of Study 2 was to further investigate the psychometric properties of the Cross-cultural Competence Inventory. While in Study 1 we only examined the internal consistency, factorial structure and theoretical validity of CCCI, in Study 2 we additionally examined the test-retest reliability, theoretical validity, criterion validity and convergent validity.

To fulfil these goals and examine the test-retest reliability, a new pool of participants completed the CCCI during two sessions separated in time. Second, to examine the criterion validity we correlated the CCCI with another scale measuring a similar theoretical concept: cultural intelligence [35]. Third, we chose a set of measurement tools to analyze the convergent validity. More precisely, as mentioned in the introduction, cross-cultural competencies may be related to other variables. Thus, in Study 2 we additionally examined the relationship between CCCI and other variables such as personality, emphatic sensitiveness, the need for cognitive closure, emotional intelligence, self-esteem and social desirability. We hypothesized, in general, that while emotional intelligence, empathy and self-esteem should be significantly positively correlated with cross-cultural competencies, the need for cognitive closure should be negatively related to these competencies. Finally, to further examine the theoretical validity, the CCCI was completed by professional cross-cultural competence trainers. We expected them to differ significantly from the non-professional group of participants; namely, we expected them to obtain higher CCCI scores than non-professionals.

### Participants

A total of 347 individuals aged 18–53 participated in the study (311 female, 36 male) (*M* = 21.49, *SD* = 4.73). All indicated Polish nationality. As recommended by the Author of the original scale [20,21], from CCCI score analysis we excluded 30 and 43 participants who scored higher than 15 on the “Lie and Social Desirability” scale in the first or second session, respectively. Finally, the 30 participants who scored higher than 15 on this scale in both sessions were excluded from the total pool of participants. Therefore, the final sample consisted of 317 participants (284 female, 33 male) aged 18–53 (*M* = 21.46, *SD* = 4.66). The majority of participants (306, around 97%) were healthcare professionals (e.g. nurse), student nurses or medical students.

Participants completed two sessions, each one on separate days (average interval = 28.06 ±4.34 days, range = 22 to 47 days). To keep the sessions as comparable as possible in terms of the time of the day and the activities undertaken, the second session was scheduled at least 22 days after the first session, at the same time and on the same day of the week as the previous one whenever possible. It should be noted that 15 participants completed only one session, but their partial data were still analyzed. No incentive was offered for participation in this two-session study.

Finally, a total of 36 professional cross-cultural competence trainers participated in the one-session study (26 females, 10 males) aged 28–65 (*M* = 45.66, *SD* = 8.61, two participants did not indicate their age). All participants finished the 250-hour Training the Trainers in Multicultural Education and Competences course offered by the Polish Helsinki Human Rights Foundation (HHRF), whose trainers are officially recommended by the HHRF as professional cross-cultural competence trainers. While we excluded 5 participants due to their score on the “Lie and Social Desirability” scale, the final sample consisted of 31 individuals (21 female, 10 male) aged 28–65 (*M* = 44.52, *SD* = 8.51).

### Materials

#### The Cross-Cultural Competence Inventory

We used the same version of the CCCI as in Study 1.

#### The Cultural Intelligence Scale

The Cultural Intelligence Scale (CQS) was developed by Ang et al. [35] to measure CQ, which is defined as the “capability to function effectively in culturally diverse settings”. The CQS consists of the four CQ dimensions: (1) metacognitive CQ (the extent to which individuals use cognitive processes in order to acquire and understand cultural knowledge); (2) cognitive CQ (explicit knowledge about practices, norms, and conventions in different cultures); (3) motivational CQ (the extent to which an individual is willing to learn about cultural differences and function in culturally diverse contexts); and (4) behavioral CQ (the extent to which individuals are capable of behaving appropriately when interacting with culturally diverse people). The CQS consists of 20 items and has satisfactory reliability coefficients ranging from 0.79 to 0.90. The Polish adaptation of the CQS is described elsewhere (Barzykowski, Majda, Przyłęcki, & Szkup, in preparation).

#### The Emphatic Sensitiveness Scale

The Emphatic Sensitiveness Scale (ESS) [39] consists of 28 items and is based on the empathy model proposed by Davis [46,47]. The ESS consists of three components: (1) Empathic Concern, (2) Personal Distress, and (3) Perspective Taking. While the first two relate to emotional aspects, the third relates to the cognitive aspect of empathy. The reliability coefficients (internal consistency) for the ESS range from .74 to .78 and the theoretical validity is confirmed.

#### The Short version of the Need For Cognitive Closure Scale

The Short version of the Need for Cognitive Closure Scale (SNCCS) [36] consists of 15 items and is based on the Need for Closure Scale by Webster and Kruglanski [48,49]. It consists of 4 domains: (1) Predictability (preference for order and structure); (2) Ambiguity (discomfort associated with the absence of closure); (3) Closed Mindedness (avoidance of alternative opinions and inconclusive evidence); and (4) Decisiveness (desire to reach closure by making judgments or decisions). The reliability coefficients (internal consistency) for the SNCCS range from .52 to .86 and the theoretical validity is confirmed.

#### The International Personality Item Pool–Big Five Markers-20

The International Personality Item Pool–Big Five Markers 20 (IPIP-20) [43] measures the Big Five personality traits: (1) Extraversion, (2) Agreeableness, (3) Consciousness, (4) Emotional Stability, (5) Intellect. The IPIP-20 has sufficient and satisfactory reliability coefficients ranging from .61 to .82 and the theoretical validity is confirmed.

#### The Social Desirability Scale

The Social Desirability Scale [50] consists of 29 items of the “true-false” type. The reliability coefficients (internal consistency and stability) for the questionnaire range from 0.79 to 0.90. High coefficients of correlation (up to 0.82) with Marlowe-Crowne's scale [51] were also obtained [50]. In this way we wanted to control for the possibility that participants deliberately tried to express their open attitudes towards culturally diverse people to please the experimenter. Social desirability is the need to be accepted and being ready to behave in a manner that is perceived favorably by others. The issue of the need for social approval bears on the majority of interviews, especially if they concern issues important to the respondent, e.g. attitude towards people from other cultures and religions.

#### The Emotional Intelligence Scale

The Emotional Intelligence Scale (EIS) [40] consists of 25 items measuring the concept of emotional intelligence introduced by Saloveya, Mayera et al. [52]. It consists of three main domains: (1) Perception of emotions and empathy (ability to recognize, identify and empathize with emotional states expressed by others); (2) Insight with emotional knowledge (insight into one’s own emotions); and (3) Mood managing (ability to manage negative emotions and states). The EIS has sufficient and satisfactory reliability coefficients ranging from .63 to .81 and the theoretical validity is confirmed.

#### The Rosenberg Self-Esteem Scale

The Polish Rosenberg Self-Esteem Scale (SES) [41] consists of 10 items and measures global self-esteem, defined as attitude towards the self. The SES has sufficient and satisfactory reliability coefficients ranging from .81 to .83 and the theoretical validity is confirmed.

#### The International Personality Item Pool–Big Five Markers 50

The International Personality Item Pool–Big Five Markers 50 (IPIP-50) [42] is the Polish adaptation of Goldberg’s IPIP-BFM-50 questionnaire for measuring the five personality traits: (1) Extraversion, (2) Agreeableness, (3) Conscientiousness, (4) Emotional Stability, and (5) Intellect. It consists of 50 items. The IPIP-50 has sufficient and satisfactory reliability coefficients ranging from .77 to .88 and the theoretical validity is confirmed.

#### Procedure

Participants were tested in groups. They were informed that they were free to withdraw from the study at any point. The interviewer assured them that their responses would be anonymous, and they could refrain from reporting particularly sensitive information by marking “X” as an answer.

In the first session, participants completed the following questionnaires: CCCI, CQS, The Emphatic Sensitiveness Scale, The Need for Closure Scale, The International Personality Item Pool–Big Five Markers 20, and The Social Desirability Scale. During the second session they completed the CCCI, The Emotional Intelligence Scale, The Rosenberg Self-Esteem Scale, and IPIP-BFM-50. Finally, the group of professional cross-cultural trainers completed only the CCCI and CQS.

### Results

#### Descriptive results and reliability: Internal consistency, test-retest reliability

The overall means for the CCCI are presented in Table 1. As can be seen, the internal consistency of the CCCI inventory (Cronbach's α) was .83 and .86 in the first and second session, respectively. Importantly, the internal consistency parameter ranged from .56 to .86 and .67 to .90 across the subscales in the first and second session, respectively. The one-month test-retest reliability (Pearson’s correlation coefficient) for the total score in CCCI was *r*(258) = .79, *p* < .001, and it ranged from .58 to .79 across the subscales.

#### Validity: Criterion, theoretical and convergent

**Criterion validity.** In order to further examine the CCCI’s criterion validity we correlated the total score of the CCCI with another tool constructed to measure a similar concept: the cultural intelligence scale (CQS) [35]. As presented in Table 3, the Pearson’s correlation coefficient between CCCI and CQS was *r*(301) = .67, *p* < .001 and *r*(31) = .76, *p* < .001, in the non-professional group and professional trainers, respectively.

**Table 3:** Correlations of the CCCI with the Cultural Intelligence Scale and other measures (e.g. personality, empathy) in Study 2.

		Study 2										
		Non-Professionals: Group of non-cross-cultural trainers							Professionals: Group of cross-cultural trainers			
		Session 1: Test			Session 2: Retest			Correlations with CCCI (total score)				Correlations CCCI
		*M*	*SD*	*Cronbach's α*	*M*	*SD*	*Cronbach's α*	*r*	*M*	*SD*	*Cronbach's α*	*r*
Cultural Intelligence Scale		81.44	19.77	.94	-	-	-	*r*(301) = .67, *p < .001	97.90	17.32	.95	*r*(31) = .76, *p < .001
Emphatic Sensitiveness Scale	Empathic Concern	40.10	5.26	.73	-	-	-	*r*(301) = .07, *p* = .211	-	-	-	-
	Personal Distress	24.53	4.40	.66	-	-	-	*r*(301) = -.31, *p < .001	-	-	-	-
	Perspective Taking	33.20	4.19	.69	-	-	-	*r*(301) = .42, *p < .001	-	-	-	-
The Need for Closure Scale	Order	12.38	2.94	.76	-	-	-	*r*(301) = -.20, *p < .001	-	-	-	-
	Predictability	163.18	1.88	.73	-	-	-	*r*(301) = -.30, *p < .001	-	-	-	-
	Ambiguity	13.14	2.47	.67	-	-	-	*r*(301) = -.34, *p < .001	-	-	-	-
	Closed Mindedness	7.79	2.21	.68	-	-	-	*r*(301) = -.53, *p < .001	-	-	-	-
	Decisiveness	10.09	3.24	.78	-	-	-	*r*(301) = .34, *p < .001	-	-	-	-
International Personality Item Pool–Big Five Markers 20	Extraversion	15.53	3.67	.82	-	-	-	*r*(301) = .33, *p < .001	-	-	-	-
	Agreeableness	18.18	2.35	.66	-	-	-	*r*(301) = .28, *p < .001	-	-	-	-
	Conscientiousness	15.27	3.39	.75	-	-	-	*r*(301) = .08, *p* = .170	-	-	-	-
	Emotional Stability	13.13	3.08	.73	-	-	-	*r*(301) = .36, *p < .001	-	-	-	-
	Intellect	16.97	2.52	.65	-	-	-	*r*(301) = .49, *p < .001	-	-	-	-
The Social Desirability Scale		15.29	4.61	.72	-	-	-	*r*(301) = .18, *p = .002	-	-	-	-
Emotional Intelligence Scale	Perception of emotions and empathy	-	-	-	34.50	4.42	.75	*r*(274) = .37, *p < .001	-	-	-	-
	Insight with emotional knowledge	-	-	-	36.21	4.00	.68	*r*(274) = .24, *p < .001	-	-	-	-
	Mood managing	-	-	-	27.69	3.78	.73	*r*(274) = .47, *p < .001	-	-	-	-
The Rosenberg Self-Esteem Scale		-	-	-	19.38	5.01	.86	*r*(274) = .30, *p < .001	-	-	-	-
IPIP-50	Extraversion	-	-	-	3.34	.74	.90	*r*(274) = .36, *p < .001	-	-	-	-
	Agreeableness	-	-	-	3.91	.52	.82	*r*(274) = .34, *p < .001	-	-	-	-
	Conscientiousness	-	-	-	3.50	.58	.81	*r*(274) = .13, *p = .029	-	-	-	-
	Emotional Stability	-	-	-	2.83	.69	.88	*r*(274) = .27, *p < .001	-	-	-	-
	Intellect	-	-	-	3.57	.51	.76	*r*(274) = .49, *p < .001	-	-	-	-

Notes: Significant results are marked with an asterisk (e.g. *p), *r*, Pearson’s correlation coefficient.

**Convergent validity.** As demonstrated in Table 3, CCCI positively correlated with (1) emphatic sensitiveness–perspective taking; (2) need for cognitive closure–decisiveness; (3) social desirability; (4) emotional intelligence–perception of emotions and empathy, insight with emotional knowledge and mood managing; and (5) self-esteem. At the same time, it was negatively correlated with (1) emphatic sensitiveness–personal distress; (2) need for cognitive closure–need for order, predictability, tolerance ambiguity, and closed mindedness. Finally, cross-cultural competencies positively correlated with personality traits such as extraversion, agreeableness, emotional stability and intellect.

**Theoretical validity.** To test the differences between professionals and non-professionals in CCCI, the overall means for the CCCI total score as well as for the CCCI’s subscales were entered into an independent *t-test*. With *α* = .05, the critical corrected value *q* was .031. As can be seen in Table 1, the professional cross-cultural trainers scored significantly higher than non-professional participants on the CCCI total score (a large effect size). More precisely, they also scored higher on the following subscales: (a) cultural adaptability (medium effect size), (b) determination (large effect size), (c) tolerance (large effect size). In addition, while the professionals scored lower on the Self-Presentation scale (medium effect size), there were no differences between groups in the mission focus and engagement scale results.

### Discussion

The main goal of Study 2 was to further examine the psychometric properties of the CCCI. More precisely, we provided additional evidence for the test-retest reliability and theoretical, criterion and convergent validity. Importantly, the CCCI has good internal consistency and test-retest reliability. In addition, it substantially correlates with the Cultural Intelligence Scale, which confirms the criterion validity. As for theoretical validity, we were able to provide additional support demonstrating that participants who were expected to obtain a high total score in the CCCI because they were professionals in fact scored higher than non-professionals. Finally, we also demonstrated that cross-cultural competencies correlated significantly with most of the variables, including emphatic sensitiveness, need for cognitive closure (mostly negatively), emotional intelligence, self-esteem and personality traits.

Taking all these findings together, since we confirmed the reliability, theoretical, criterion and convergent validity, we feel confident that the CCCI is a reliable and valid psychological tool for measuring cross-cultural competencies. We further discuss these results in the relevant section below.

## General discussion

The basis of the Polish adaptation was the American version of the Cross-Cultural Competence Inventory (CCCI), by Thornson [20], Thornson & Ross [21]. The psychometric characteristics of the original questionnaire were satisfactory. For this reason, we translated the CCCI into Polish.

Next, across two studies we examined the psychometric properties of the Polish version of the CCCI: (1) reliability–internal consistency, factor structure, test-retest reliability; (2) validity–theoretical, criteria, convergent. We discuss these properties in the relevant sections below.

### The general reliability of the CCCI: Internal consistency, factorial structure, test-retest reliability

The first goal of the present study was to analyze the reliability of the CCCI. Across two studies we were able to provide evidence that the CCCI has satisfactory internal consistency and test-retest reliability. More precisely, our results suggest that cross-cultural competencies are quite stable over time. While the original version of the CCCI was assumed to have a 6-dimensional factor structure [20,21], a confirmatory factor analysis did not provide any confirmation for this assumption: this factor structure simply did not fit our empirical data well. Thus we argue that one should not strictly analyze the scores on subscales. However, since all items were significantly related to one general factor, we highly recommend the usage of the total score of the CCCI.

### The general validity of the CCCI: Theoretical, criterion and convergent validity

The second goal of the present study was to further examine the reliability of the CCCI. As can be seen in Table 3, the CCCI highly correlated with another tool constructed to measure the theoretically similar concept of cultural intelligence. This provides evidence for satisfactory criterion validity.

Second, our findings successfully confirmed the CCCI’s theoretical validity consistently across two studies. As expected, the professional cross-cultural trainers scored significantly higher on the CCCI than non-professionals. In addition, respondents who had had an encounter with members of cultural or religious minorities had a higher level of cultural competence. Importantly, negative experiences did not seem to affect these competences because people with bad experiences did not differ in terms of CCCI. Respondents who declared a positive attitude to culturally diverse national and religious groups of people achieved significantly higher scores in the CCCI. These results replicate the findings of, for example, Czerniejewska [53], Branka and Cieślikowska [54]. It can be argued that contact with culturally divergent people may be an important element of interpersonal and social development. For instance, our intercultural sensitivity develops thanks to communication with culturally diverse individuals; thus, we may better get to know ourselves and our identity. At the same time, negative attitudes towards cultural diversity may close us into a system of only one (i.e. our own) set of cultural values, thus making us accept only our own perspective of the world. As a result, negative attitudes deprive us of a chance to develop. At the same time, establishing creative relationships with members of new intercultural groups breaks the ethnocentric point of view, according to which anything that is culturally divergent is perceived as inferior compared to the norm adopted by our own culture. Such ethnocentrism is expressed as a feeling of superiority in which our own culture, its principles, norms, and values is treated as the only right one, while all others are perceived as inferior. While the tendency to evaluate culturally divergent people from the perspective of one’s own cultural standards is common, it hinders contact with Others. Intercultural relations become creative when one adopts an attitude relating to cultural relativism in which behavior, values, and norms are interpreted in the context of a particular culture. This minimizes the tendency to judge and consider cultural differences as good or bad. One may simply perceive them as ‘different’, but not inferior. Cultural relativism fosters the development of cognitive curiosity, expressed in, for example, the desire to learn about the Other. Thanks to this, we are able to perceive Others as interesting and valuable partners–an attitude that enables good cross-cultural encounters.

Finally, in study 2 we also provided evidence for the convergent validity of the CCCI. More precisely, cross-cultural competencies significantly correlated with some variables: (1) personality–extraversion, agreeableness, emotional stability, intellect (positive correlation); (2) empathic sensitivity–taking perspective (positive correlation), personal suffering (negative correlation); (3) the need for cognitive closure–decisiveness (positive correlation) and the need for order, predictability, ambiguity of tolerance, closed mind (mainly negative correlation); (4) emotional intelligence–emotion perception and empathy, insight into management of emotional knowledge and mood (positive correlation); (5) self-esteem (positive correlations); (6) social approval (positive correlations). These results additionally replicate findings from the literature showing the relationship between cross-cultural competencies and the other factors mentioned above [37,38,55,56]. For instance, as expected, the need for cognitive closure negatively correlated with cultural competences: people with a high need for closure prefer order, like to be predictable, are cognitively closed and have less tolerance for uncertainty. As expected, other variables such as personality, emotional intelligence, and self-esteem correlated positively with cultural competences. As for personality, we observed significant positive correlations between cross-cultural competency and stable personality traits such as extraversion, intellect, emotional stability and agreeableness. These results are in line with findings reported in the literature [57] and suggest that they may be inherent in cross-cultural competencies [58]. For instance, as argued by Caligiuri and Tarique [59], intellect (which relates to an individual’s curiosity, willingness to take risks and openness to new and novel experiences) and extraversion ‘may predispose individuals to seek out experiences and interact with people from different cultures’ and, importantly, may also enhance their motivation to learn [60]. Thus, someone with high intellect, extraversion, emotional stability (individuals higher in emotional stability are less likely to be anxious, emotional, worried or angry) and self-esteem would be more prone to voluntarily engage in cross-cultural encounters and, importantly, would be also more ready to interact with culturally diverse individuals when the opportunity arises. Such a constellation of personality traits (i.e. high extraversion, intellect, emotional stability and self-esteem) is a good predictor of tolerance of ambiguity and cultural flexibility [59,61], defined as ‘the capacity to substitute activities enjoyed in one’s home country with existing, and usually distinct, activities in the host country’ [62] As a result of having more multicultural experiences, ethnocentrism may also be more efficiently reduced. Finally, we also observed a positive relationship between emphatic sensitiveness, agreeableness (the extent to which an individual is warm, tactful, friendly and tolerant [62]) and cross-cultural competencies. These characteristics may predispose an individual to correctly recognize and acknowledge someone’s emotions and to adapt their own behavior accordingly. As a result, trustful and positive interpersonal cross-cultural encounters may be more frequent among those high in agreeableness and empathic sensitiveness.

In summary, all these findings together may give us an interesting insight into the factors that significantly influence cross-cultural competencies. This should be taken into careful consideration when preparing and executing cross-cultural training.

### The cross-cultural competencies inventory as an assessment instrument for healthcare professionals

It can be argued that having cultural competences is a key element in providing effective and culturally sensitive medical care to patients from culturally and/or ethnically different circles [63]. According to Hammer et al. having a basic set of cultural competences allows adaptation to any culture [64]. Due to the necessity of providing culturally sensitive care in medical practice, the measurement of competences and their impact on the quality of patient care is essential for the development of basic human care [65]. The latest research on this topic, conducted in various parts of the world, indicates the importance of cultural competences for the accumulation of intellectual capital among nurses (Taiwan) [66]. These competences are also important in the context of the preparation of nursing students for their professional roles in the future (e.g. Saudi Arabia [67] or Philippines [68]). There are also already existing programs that aim to develop cultural competencies in undergraduate nursing students [69]. The literature contains proposals of tools that assess cultural competencies in medical care; nevertheless, reliable assessment of cultural competences among Polish medical care workers is still quite difficult, mainly due to the lack of validation and adaptation of the scales to Polish conditions. The CCCI seems to be an appropriate tool that can assess the possibility of the effective adaptation of health care professionals to providing care for patients from other cultures. In summary, we argue that the CCCI may be a useful tool in evaluating cultural competencies of health care professionals and students of, for example, medical faculties. Thus, it may be possible to identify strengths and weaknesses in cultural competences and to plan the right actions for future professional self-improvement.

### Possible limitations and future directions

When considering the results of the present study, some limitations should be taken into account. First, it may be argued that the CCCI in its current form is quite long and completing it seems time consuming. For this reason, there is a need to develop a short version of the CCCI that preserves satisfactory reliability and validity. Second, the biggest limitation of the present study is the fact that the CFA did not confirm the 6-dimensional structure of the CCCI. Thus, we recommend using the total score of the CCCI, especially because all items are significantly related to one general factor. At the same time, while a thorough examination of the factor structure of the Polish version of the CCCI exceeds the scope of the present paper, this is already one of the most important issues that needs to be addressed in future studies. For instance, this could be achieved while thoroughly analyzing the structure of the CCCI in order to develop a short version containing the strongest and most powerful items. This would definitely extend the possibilities of the potential practical applications of this method. Third, it is worth noting that all measures reported in the present studies were based on self-report, which may limit the conclusions that can be drawn. For example, it may be possible that participants declared having a more positive attitude towards cultural diversity than they really had. This limitation is supported by the observed positive weak but significant correlation between social desirability and the CCCI. However, in order to avoid this type of bias we reassured participants about the anonymity of their responses. We also excluded from the analysis participants who scored high on the Lie and Social Desirability scale. In addition, it is also unknown to what extent the self-reports correspond to real-life behavior. Therefore, future studies could verify how differences in CCCI are reflected by real-life behaviors (e.g. the way an individual interacts with culturally diverse patients). Finally, it would also be beneficial to further study the relationship between the cross-cultural competencies measured by the CCCI and other variables (e.g. mindfulness) that may significantly influence this important professional competence. For instance, people with elevated mindfulness indicators perceive images that are usually ignored by inattentive persons [64]. Mindfulness can therefore be useful in intercultural communication [38,55] and should therefore positively correlate with cultural competences. Finally, it would be also beneficial to further study the relationship between the cross-cultural competencies measured by the CCCI and other variables (e.g. mindfulness) that may significantly influence this important professional competence. For instance, people with elevated mindfulness indicators perceive images that are usually ignored by inattentive persons [70]. Mindfulness can therefore be useful in intercultural communication [38,55] and should therefore positively correlate with cultural competences. Finally, the CCCI might be also used within different groups of participants (i.e. teachers) to evaluate cross-cultural competencies among different types of professionals. As is evident from the foregoing discussion, the results open up a set of questions for future research.

### Final conclusions

It can be argued that validating tools that measure cultural competencies may be considered an important objective for contemporary research, especially in light of the current processes of globalization and migration that affect everyone’s everyday social reality. Such objectives are particularly important for the development of research on cultural competencies in Poland. More precisely, in the relatively homogeneous Polish society, meeting the needs of culturally diverse patients is sometimes a neglected area, both in the education of health care workers and in the sphere of research. With the introduction of new educational standards in 2012, the legal situation in the education of students at medical faculties began to change; this gave medical universities the opportunity to introduce to medical curricula content related to intercultural communication. There is still a need to invest in the development of cultural competences and their measurement with standardized tools. Despite the possible limitations of the presented tool, the CCCI should be considered an important and reliable tool for assessing cultural competences among healthcare professionals (e.g. doctors, nurses) and students. Importantly, as demonstrated in the two studies described, the CCCI has satisfactory psychometric properties. More precisely, we conclude that our psychometric validation of the CCCI allowed us to prove that the Polish version of it is reliable and valid questionnaire. We postulate that the CCCI scale can be used in a practical way by nursing educators and clinical managers in the separation of areas of cultural competence, the development of which is necessary to improve the quality of healthcare. The use of the CCCI could also be helpful in choosing the content of intervention programs aimed at minimizing competence shortages and assessing their effectiveness in order to improve the cultural competence of healthcare professionals. This competence is already well recognized as a crucial element in providing culturally competent patient-centered care (e.g. [69,71,72]). Conducting empirical research with the CCCI will make it possible to observe the development of the cultural competencies of medical students and to independently improve their intercultural education. While there is still a need to analyze how such culturally competent patient-centered care may influence the quality of healthcare [73], it may be reasonably argued that it already contributes to meeting the challenge of providing excellent care that improves the satisfaction, well-being and health outcomes of patients.

## Supporting information

S1 Appendix The Polish version of the Cross-Cultural Competence Inventory. (PDF)

S1 Dataset Data base of Study 1 and Study 2. (ZIP)

## References

[1] Eurostat. Foreign born population by country of birth. 2018. Available from: https://ec.europa.eu/eurostat/statistics-explained/index.php?title=File:Foreign-born_population_by_country_of_birth,_1_January_2017_.png

[2] Szczepanik M. Cudzoziemcy w Polsce—zjawiska i charakterystyka kulturowa wybranych grup In: Ostaszewska-Żuk E, editor. Cudzoziemcy w Polsce. Podręcznik dla funkcjonariuszy publicznych. Warszawa: Helsińska Fundacja Praw Człowieka; 2016 pp. 13–34.

[3] Główny Urząd Statystyczny. Szkolnictwo wyższe w roku akademickim 2017/2018 (dane wstępne). Available from: https://stat.gov.pl/obszary-tematyczne/edukacja/edukacja/szkolnictwo-wyzsze-w-roku-akademickim-20172018-dane-wstepne,8,5.html

[4] Internetowy System Aktów Prawnych. Rozporządzenie Ministra Nauki i Szkolnictwa Wyższego z dnia 9 maja 2012 r. w sprawie standardów kształcenia dla kierunków studiów: lekarskiego, lekarsko-dentystycznego, farmacji, pielęgniarstwa i położnictwa, Dz.U.2012 poz. 631. Available from: http://prawo.sejm.gov.pl

[5] Leininger M. Overview of the theory of culture care with the ethnonursing research method. J Transcult Nurs. 1997;8(2): 32–52. 10.1177/104365969700800205 9369663

[6] Leininger MM. Culture care diversity and universality: A theory of nursing New York: John Wiley&Sons; 1991.

[7] Schim SM, Doorenbos AZ, Benkert R, Miller J. Culturally congruent care: Putting the puzzle together. J Transcult Nurs. 2007;18(2): 103–110. 10.1177/1043659606298613 17416711

[8] Campinha-Bacote J. Many Faces: Addressing Diversity in Health Care. Online J Issues Nurs. 2003;8(1). Available from: http://www.nursingworld.org 12729453

[9] Campinha-Bacote J. The Process of Cultural Competence in the Delivery of Healthcare Services: The journey continues. Cincinnati, Ohio: Transcultural C.A.R.E. Associates; 2007.

[10] Purnell L. The Purnell Model for Cultural Competence. J Transcult Nurs. 2002;13(3): 193–196. 10.1177/10459602013003006 12113149

[11] Purnell LD, Paulanka BJ. Guide to culturally competent health care. 2th ed Philadelphia: F.A. Davis Company; 2009.

[12] Giger JN, Davidhizar R. Transcultural nursing: Assessment and intervention. 4th ed. St. Louis: Mosby; 2004.

[13] Andrews MM. Theoretical Foundations of Transcultural Nursing In: Andrews MM, Boyle JS, editors. Transcultural Concepts in Nursing Care. 3rd ed. Philadelphia: Lippincott; 1999.

[14] Loftin C, Hartin V, Branson M, Reyes H. Measures of Cultural Competence in Nurses: An Integrative Review. ScientificWorldJournal. 2013; 2013:289101 10.1155/2013/289101 23818818 PMC3683494

[15] Perng S, Watson R. Construct validity of the nurse cultural competence scale: a hierarchy of abilities. J Clin Nurs. 2012;21(11–12): 1678–84. 10.1111/j.1365-2702.2011.03933.x 22239136

[16] Rew L, Becker H, Cookston J, Khosropour S, Martinez S. Measuring cultural awareness in nursing students. J Nurs Educ. 2003;42(6): 249–257. 12814215 10.3928/0148-4834-20030601-07

[17] Campinha-Bacote J. Reported reliability and validity of the IAPCC-R. 2009 Available from: http://www.transculturalcare.net/iapccr.htm; on-line Access 15 Jun 2018. Campinha-Bacote J. Reported reliability and validity of the IAPCC-R. 2009, http://www.transculturalcare.net/iapcc-r.htm.

[18] Sealey LJ, Burnett M, Johnson G. Cultural competence of baccalaureate nursing faculty: are we up to the task? J Cult Divers. 2006;13(3): 131–140. 16989249

[19] Brathwaite AC, Majumdar B. Evaluation of a cultural competence educational programme. J Adv Nurs. 2006;53(4): 470–479. 10.1111/j.1365-2648.2006.03742.x 16448490

[20] Thornson CA. Development and validation of the Cross-cultural Competence Inventory. Electronic Theses and Dissertations Paper 1685, University of Central Florida Libraries. 2010. Available from: http://library.ucf.edu Cited 07 Jun 2018.

[21] Thornson CA, Ross KG. The Construct & Criterion Validation of the Cross-Cultural Competence Inventory Final Report. Defense Equal Opportunity Management Institute (DEOMI); 2010.

[22] Matsumoto D, Hwang HC. Assessing cross-cultural competence: a review of available tests. J Cross Cult Psychol. 2013;44(6): 849–873.

[23] Bernhard G, Knibbe RA, von Wolff A, Dingoyan D, Schulz H, Mősko M. Development and Psychometric Evaluation of an Instrument to Assess Cross-Cultural Competence of Healthcare Professionals (CCCHP). PLoS One. 2015;10(12): e0144049 10.1371/journal.pone.0144049 26641876 PMC4671537

[24] Matveev AV, Merz MY. Intercultural Competence Assessment: What Are Its Key Dimensions Across Assessment Tools? International Association for Cross-Cultural Psychology Conferences. 2014. Available from: http://www.iacp.org/sites/default/files/stellenbosch_pdf/Matveev.pdf Cited 03 Aug 2018.

[25] Szkup-Jabłońska M, Schneider-Matyka D, Kubiak J, Grzywacz A, Jurczak A, Augustyniak K, et al Ocena kompetencji kulturowych pracowników ochrony zdrowia. Fam Med Prim Care Rev. 2013;15, 394–396.

[26] Zdziebło K, Nowak-Starz G, Makieła E, Stępień R, Wiraszka G. Kompetencje międzykulturowe w pielęgniarstwie. Probl Pielęg. 2014;22(2): 367–372.

[27] Dobrowolska B, Ozga D, Gutysz-Wojnicka A, Zeleníková R, Jarošová D, Nytra I, et al Kompetencje i potrzeby edukacyjne pielęgniarek OIT w zakresie opieki wielokulturowej. Raport projektu: 2016-1-PL01-KA202-026615. 8 2017 Available from: http://mice-icu.eu/wp-content/uploads/2017/12/O1-ICU-Nurses-intercultural-training-needs-and-competencies-analysis-report_PL.pdf Cited 25 Aug 2018.

[28] Lubowiecki-Vikuk A, Gnusowski M. Rola kompetencji międzykulturowych na rynku turystyki medycznej w Polsce. Hyg Pub Health. 2016;51(3): 255–261.

[29] Majda A, Zalewska-Puchała J. Wrażliwość międzykulturowa w opiece pielęgniarskiej. Probl Pielęg. 2011;21(3): 327–334.

[30] Majda A, Zalewska-Puchała J. Kulturowe odrębności w pielęgniarstwie In: Zarzycka D, Ślusarska B, editors. Podstawy pielęgniarstwa. Założenia koncepcyjno-empiryczne opieki pielęgniarskiej. Warszawa: WL PZWL; 2017 pp. 119–140.

[31] Majda A, Zalewska-Puchała J. Kompetencje kulturowe i inteligencja kulturowa w pielęgniarstwie. Pielęg Pol. 2018;2(68): 196–303.

[32] Mroczkowska R. Odmienność kulturowa jako nowe wyzwanie w praktyce pielęgniarki i położnej. Pielęg Spec. 2013;1: 27–31.

[33] Ślusarska B, Zarzycka D, Majda A, Dobrowolska B. Kompetencje kulturowe w pielęgniarstwie–podstawy konceptualizacji i narzędzia pomiaru naukowego. Pielęg XXI w. 2017;4(61): 40–45.

[34] Arredondo P, Toporek R, Brown S, Jones J, Locke DC, Sanchez J, et al Operationalization of the Multicultural Counseling Competencies. J Multicult Couns Devel. 1996;24(1): 42–78.

[35] Ang S, Van Dyne L, Koh C, Ng KY, Templer KJ, Tay C, et al Cultural intelligence: Its measurement and effects on cultural judgment a decision making, cultural adaptation and task performance. Manag Organiz Rev. 2007;3: 335–371.

[36] Kossowska M, Hanusz K, Trejtowicz M. Skrócona wersja Skali Potrzeby Poznawczego Domknięcia. Dobór pozycji i walidacja skali. Psychol Społ. 2012;1(20): 89–99.

[37] Szopski M. Komunikowanie międzykulturowe. Warszawa: WSiP; 2005.

[38] Matsumoto D, Juang L. Psychologia międzykulturowa. Gdańsk: GWP; 2007.

[39] Kaźmierczak M, Plopa M, Retowski S. Skala Wrażliwości Empatycznej. Prz Psychol. 2007;50(1): 9–24.

[40] Borkowska A, Gąsiorowska A, Nosal CS. Kwestionariusz do diagnozy poziomu inteligencji emocjonalnej. Prz Psychol 2006;49(1): 9–20.

[41] Łaguna M, Lachowicz-Tabaczek K, Dzwonkowska I. Skala samooceny SES Morrisa Rosenberga–polska adaptacja metody. Psychol Społ. 2007;02(04): 164–176.

[42] Strus W, Cieciuch J, Rowiński T. Polska adaptacja kwestionariusza IPIP-BFM-50 do pomiaru pięciu cech osobowości w ujęciu leksykalnym. Rocz Psychol. 2014;XVII(2): 327–346.

[43] Topolewska E, Skimina E, Struś W, Cieciuch J, Rowiński T. Krótki Kwestionariusz do Pomiaru Wielkiej Piątki IPIP-BFM-20. Rocz Psychol. 2014;XVII(2): 367–384.

[44] Benjamini Y, Hochberg Y. Controlling the false discovery rate: A practical and powerful approach to multiple testing. J R Stat Soc Series B Stat Methodol. 1995;57: 289–300.

[45] Cohen J. Statistical power analysis for the behavioral sciences. 2nd ed. New York: Erlbaum; 1988.

[46] Davis MH. Measuring individual differences in empathy: Evidence for a multidimensional approach. J Pers Social Psychol. 1983;44(1): 113–126.

[47] Davis MH. Empatia. O umiejętności współodczuwania. Gdańsk: GWP; 1999.

[48] Webster D, Kruglanski AW. Individual differences in need for cognitive closure. J Pers Social Psychol. 1994;67: 1049–1062. 10.1037//0022-3514.67.6.1049 7815301

[49] Kossowska M. Różnice indywidualne w potrzebie poznawczego domknięcia. Prz Psychol. 2003;46:355–375.

[50] Drwal RŁ, Wilczyńska IT. Opracowanie Kwestionariusza Aprobaty Społecznej. Prz Psychol. 1980;23: 569–583.

[51] Crowne DP, Marlowe D. A new scale of social desirability independent of psychopathology. J Consult Psychol. 1960;24(4): 349–354. 13813058 10.1037/h0047358

[52] Salovey P, Mayer JD, Goldman SL, Turvey C, Palfai T P. Emotional attention, clarity and repair: Exploring emotional intelligence using the Trait Meta-Mood Scale In: Pennebaker JW, editor. Emotion, disclosure and health. Washington: American Psychological Association; 1995.

[53] Czerniejewska I, editor. Antydyskryminacja na co dzień. Poznań: Stowarzyszenie Jeden Świat; 2005.

[54] Branka M, Cieślikowska D, editors. Edukacja antydyskryminacyjna. Kraków: Willa Decjusza; 2010.

[55] Ting-Toomey S. Communicating Across Cultures.New York: Guilford Press 1999.

[56] Barna LM. Stumbling blocks in intercultural communication In: Samovar LA, Porter RE. editors. Intercultural communication: A reader. 8th ed. Belmont, CA: Wadsworth; 1996 pp. 370–379.

[57] Wilson J, Ward C, Fischer R. Beyond Culture Learning Theory: What Can Personality Tell Us About Cultural Competence? J Cross Cult Psychol. 2013;44(6): 900–927.

[58] Caligiuri P, Mencin A, Jayne B, Traylor A. Developing cross-cultural competencies through international corporate volunteerism. J World Bus. 2019;54(1): 14–23.

[59] Caligiuri, Tarique I. Dynamic cross-cultural competencies and global lea- dership effectiveness. J World Busi 2012;47: 612–622.

[60] Major DA, Turner JE, Fletcher TD. Linking proactive personality and the Big Five to motivation to lean and development activity. J Appl Psychol. 2006; 91: 927–935. 10.1037/0021-9010.91.4.927 16834515

[61] Mendenhall M, Oddou G. The dimensions of expatriate acculturation: A review. Acad Manage Rev. 1985;10: 39–47.

[62] Shaffer M, Harrison D, Gregersen H, Black S. (2006). You can take it with you: Individual differences and expatriate effectiveness. J Appl Psychol. 2006;91: 109–125. 10.1037/0021-9010.91.1.109 16435942

[63] Earley PC, Ang S. Cultural Intelligence: Individual interactions across cultures Stanford: Stanford University Press; 2003.

[64] Hammer MR. Behavioral dimensions of intercultural effectiveness: A replication and extension. Int J Intercult Relat. 1987;11: 65–88.

[65] Goldberg LR. An alternative description of personality: The Big Five factor structure. J Pers Soc Psychol. 1990;59(6): 1216–1229. 2283588 10.1037//0022-3514.59.6.1216

[66] Lin HC. Impact of nurses' cross-cultural competence on nursing intellectual capital from a social cognitive theory perspective. J Adv Nurs. 2016;72(5): 1144–54. 10.1111/jan.12901 26786200

[67] Cruz JP, Alquwez N, Cruz CP, Felicilda-Reynaldo RFD, Vitorino LM, Islam SMS. Cultural competence among nursing students in Saudi Arabia: a cross-sectional study. Int Nurs Rev. 2017;64(2): 215–223. 10.1111/inr.12370 28295279

[68] Cruz JP, Estacio JC, Bagtang CE, Colet PC. Predictors of cultural competence among nursing students in the Philippines: A cross-sectional study. Nurse Educ Today. 2016;46: 121–126. 10.1016/j.nedt.2016.09.001 27636832

[69] Cupelli L. An innovative service-learning project to develop cultural competency in undergraduate nursing students. Teaching and Learning in Nursing. 2016;11(3): 113–117.

[70] Radoń S. Pięciowymiarowy kwestionariusz uważności: polska adaptacja. Roczniki Psychologiczne 2014; XVII(4): 711–735.

[71] Darnell LK, Hickson SV. Cultural Competent Patient-Centered Nursing Care. Nursing Clinics of North America. 2015;50(1): 99–108. 10.1016/j.cnur.2014.10.008 25680490

[72] Mareno N, Hart PL. Cultural competency among nurses with undergraduate and graduate degrees: Implications for nursing education. Nursing Educational Perspectives. 2014;35(2): 83–88. 10.5480/12-834.1 24783722

[73] Diallo AF, McGrath JM. A Glance at the Future of Cultural Competency in Healthcare. Newborn and Infant Nursing Reviews. 2013; 13(3): 121–123.

