# Supplementary material for: The Cross-Cultural Competence Inventory: Validity and psychometric properties of the Polish adaptation
Source: PLoS One. 2019 Mar 7;14(3):e0212730. doi: 10.1371/journal.pone.0212730 (PMC6405162; doi:10.1371/journal.pone.0212730)
Supplement: S1 Appendix — (PDF) [file pone.0212730.s001.pdf]

INWENTARZ KOMPETENCJI MIĘDZYKULTUROWYCH

Pseudonim:…………………………………………………… Płeć:………………… Wiek:………………

Wykształcenie:………………………………………………… Zawód:…………………………………………….
Email/dane kontaktowe (opcjonalne): ……………………………………………. Narodowość ……………………………………

Instrukcja: Zapoznaj się uważnie z poniższymi stwierdzeniami a następnie określ, w jakim stopniu zgadasz się z każdym z nich. Do każdego stwierdzenia należy ustosunkować się korzystając z następującej skali:

Zdecydowanie nie zgadzam się					Zdecydowanie zgadzam się
1	2	3	4	5	6

Nie zastanawiaj się długo nad odpowiedzią. Zazwyczaj pierwsza odpowiedź jest najlepsza. Nie ma tutaj odpowiedzi dobrych i złych, dlatego odpowiadaj szczerze i zgodnie z własnym przekonaniem.

Zaznacz swoje odpowiedzi poprzez zakreślenie kółkiem cyfry w odpowiedniej kratce.

		Zdecydowanie nie zgadzam się					Zdecydowanie zgadzam się
1.	Nie lubię, gdy czyjaś wypowiedź może mieć wiele różnych znaczeń.	1	2	3	4	5	6
2.	Próbuję szukać logicznego wyjaśnienia lub rozwiązania prawie każdego problemu, który napotkam.	1	2	3	4	5	6
3.	Kiedy ktoś przerwie czynność, którą wykonuję, nie mam problemu z ponownym skupieniem się na niej.	1	2	3	4	5	6
4.	Podczas kontaktu z ludźmi o różnym pochodzeniu etnicznym lub kulturowym, priorytetem staje się dla mnie zrozumienie ich punktu widzenia.	1	2	3	4	5	6
5.	Lubię mieć wszystko dobrze zaplanowane i każdą rzecz na swoim miejscu.	1	2	3	4	5	6
6.	Lubię przemawiać przed grupą przyjaciół.	1	2	3	4	5	6
7.	Uważam, że jasne reguły i porządek w pracy są podstawą sukcesu.	1	2	3	4	5	6
8.	Sukces w pracy zależy w dużym stopniu od tego, czy rozumiesz się dobrze z ludźmi, z którymi pracujesz.	1	2	3	4	5	6
9.	Gdy spotykam osobę, którą znam, zazwyczaj zatrzymuję się i rozmawiam z nią.	1	2	3	4	5	6

Wersja oryginalna kwestionariusza: Thornson, C. A., & Ross, K. G. (2010, August). The Construct & Criterion Validation of the Cross-Cultural Competence Inventory: Final Report. Directed by Dr. Daniel P. McDonald, Patrick AFB, FL: Defense Equal Opportunity Management Institute (DEOMI).

Wersja polska za zgodą autorów: Barzykowski, K., Majda, A., Przyłęcki, P., & Szkup, M. (2019). The Cross-Cultural Competence Inventory: Validity and Psychometric Properties of the Polish Adaptation. PLoS ONE. DOI: 10.1371/journal.pone.0212730

		Zdecydowanie nie zgadzam się				Zdecydowanie zgadzam się
10.	Rozmawianie z osobami pochodzącymi z innych kultur sprawiałoby mi przyjemność.	1	2	3	4	5	6
11.	Nigdy nie znałem/am osoby, której nie lubiłem/am.	1	2	3	4	5	6
12.	Kiedy idę na zakupy, nie mam problemów z podjęciem decyzji odnośnie tego, co dokładnie chcę kupić.	1	2	3	4	5	6
13.	Jestem pewny/a, że potrafił(a)bym nawiązywać kontakty towarzyskie z ludźmi pochodzącymi z innej kultury.	1	2	3	4	5	6
14.	Kiedy chcę poczuć bardziej pozytywne emocje (szczęście lub radość), zaczynam myśleć o czymś innym.	1	2	3	4	5	6
15.	Czuję dyskomfort kiedy nie rozumiem dlaczego dana sytuacja przydarzyła się właśnie mnie.	1	2	3	4	5	6
16.	Nigdy nie określił(a)bym siebie jako osoby niezdecydowanej.	1	2	3	4	5	6
17.	Nie zawsze jestem taką osobą, jaką wydaję się być.	1	2	3	4	5	6
18.	Mógłbym/mogłabym zmienić sposób, w jaki mówię (np. akcent czy ton głosu), gdyby kontakt z osobą z innej kultury tego wymagał.	1	2	3	4	5	6
19.	Nigdy nie spóźniłem/am się na spotkanie.	1	2	3	4	5	6
20.	Mogę odnosić większe sukcesy w pracy, jeśli zrozumiem co jest ważne dla innych ludzi.	1	2	3	4	5	6
21.	Gdy rozwiązuję dany problem, potrafię przewidzieć odległe konsekwencje podejmowanych przeze mnie działań.	1	2	3	4	5	6
22.	Nienawidzę zmieniać moich planów w ostatniej chwili.	1	2	3	4	5	6
23.	Kontroluję moje emocje zmieniając sposób myślenia o sytuacji, w której się znalazłem/am.	1	2	3	4	5	6
24.	Moje zachowanie jest zwykle wyrazem moich prawdziwych uczuć, postaw i przekonań.	1	2	3	4	5	6

Wersja oryginalna kwestionariusza: Thornson, C. A., & Ross, K. G. (2010, August). The Construct & Criterion Validation of the Cross-Cultural Competence Inventory: Final Report. Directed by Dr. Daniel P. McDonald, Patrick AFB, FL: Defense Equal Opportunity Management Institute (DEOMI).

Wersja polska za zgodą autorów: Barzykowski, K., Majda, A., Przyłęcki, P., & Szkup, M. (2019). The Cross-Cultural Competence Inventory: Validity and Psychometric Properties of the Polish Adaptation. PLoS ONE. DOI: 10.1371/journal.pone.0212730

		Zdecydowanie nie zgadzam się					Zdecydowanie zgadzam się
25.	Jeśli moje podejście do rozwiązania danego problemu nie wpływa korzystnie na moją współpracę z kimś, z łatwością potrafię zmieniać swoją taktykę.	1	2	3	4	5	6
26.	Jeśli mam do wykonania zadanie wraz z innymi ludźmi najpierw chcę ich dobrze poznać.	1	2	3	4	5	6
27.	Nie lubię sytuacji, które są nieokreślone lub niepewne.	1	2	3	4	5	6
28.	Kontaktując się z osobami pochodzącymi z innych kultur lub grup etnicznych okazuję swoje uznanie dla ich własnych norm kulturowych.	1	2	3	4	5	6
29.	Uważam, że nie należy się angażować w rozrywkę.	1	2	3	4	5	6
30.	W różnych sytuacjach i będąc z różnymi osobami często zachowuję się jak zupełnie inna osoba.	1	2	3	4	5	6
31.	Łatwo mi zrozumieć, co czuje osoba pochodząca z obcej kultury.	1	2	3	4	5	6
32.	Nie lubię nieprzewidywalnych sytuacji.	1	2	3	4	5	6
33.	Jestem pewien/pewna swoich umiejętności w zakresie właściwego komunikowania się z różnymi osobami pochodzącymi z jakiejkolwiek grupy etnicznej i kulturowej.	1	2	3	4	5	6
34.	Uważam, że dobrze uporządkowane życie z regularnymi godzinami pracy odpowiada mojemu temperamentowi.	1	2	3	4	5	6
35.	Potrafię oszukiwać ludzi poprzez bycie miłym/miłą dla nich, choć tak naprawdę nie lubię ich.	1	2	3	4	5	6
36.	Mierząc się z sytuacją stresującą, myślę o niej w sposób, który pozwala mi zachować spokój.	1	2	3	4	5	6
37.	Podróżowanie do innych krajów to coś, co sprawiłoby mi radość.	1	2	3	4	5	6
38.	Wolę kontakty towarzyskie z osobami, które znam, ponieważ wiem czego mogę się po nich spodziewać.	1	2	3	4	5	6

Wersja oryginalna kwestionariusza: Thornson, C. A., & Ross, K. G. (2010, August). The Construct & Criterion Validation of the Cross-Cultural Competence Inventory: Final Report. Directed by Dr. Daniel P. McDonald, Patrick AFB, FL: Defense Equal Opportunity Management Institute (DEOMI).

Wersja polska za zgodą autorów: Barzykowski, K., Majda, A., Przyłęcki, P., & Szkup, M. (2019). The Cross-Cultural Competence Inventory: Validity and Psychometric Properties of the Polish Adaptation. PLoS ONE. DOI: 10.1371/journal.pone.0212730

		Zdecydowanie nie zgadzam się				Zdecydowanie zgadzam się
39.	Lubię prowadzić rozmowy w dużym gronie przyjaciół i znajomych.	1	2	3	4	5	6
40.	Kiedy myślę o problemie, rozważam tak wiele różnych możliwości jego rozwiązania, jak to tylko możliwe.	1	2	3	4	5	6
41.	Sądzę, że nie ma czegoś takiego jak niezamierzony błąd.	1	2	3	4	5	6
42.	Potrafię wygłaszać improwizowane przemówienia nawet na tematy, o których praktycznie nic nie wiem.	1	2	3	4	5	6
43.	Przyglądając się sytuacjom konfliktowym zwykle dostrzegam, że obydwie strony mogą mieć rację.	1	2	3	4	5	6
44.	Nie lubię wchodzić w sytuacje, po których nie wiem czego się spodziewać.	1	2	3	4	5	6
45.	Jeśli mam kłopot, trudno mi się skoncentrować na tym, co mam zrobić.	1	2	3	4	5	6
46.	Potrafię kontrolować moje myśli, tak żeby mnie nie rozpraszały w wykonywaniu zadania, którym aktualnie się zajmuję.	1	2	3	4	5	6
47.	Jestem pewien/a, że był(a)bym w stanie poradzić sobie ze stresem związanym z przystosowaniem się do nowej dla mnie kultury.	1	2	3	4	5	6
48.	Jeśli to konieczne, potrafię kłamać patrząc komuś prosto w oczy.	1	2	3	4	5	6
49.	Jednym z moich priorytetów jest upewnienie się, że wszyscy w moim zespole dobrze ze sobą współpracują.	1	2	3	4	5	6
50.	Kiedy stoję przed problemem zwykle potrafię znaleźć kilka jego rozwiązań.	1	2	3	4	5	6
51.	Poznawanie nowych, nieznanych dla mnie kultur, sprawiałoby mi przyjemność.	1	2	3	4	5	6
52.	Nigdy nie uraziłem/am uczuć innej osoby.	1	2	3	4	5	6
53.	Trudno mi powstrzymać myśli, które przeszkadzają mi w realizacji wykonywanego zadania.	1	2	3	4	5	6

Wersja oryginalna kwestionariusza: Thornson, C. A., & Ross, K. G. (2010, August). The Construct & Criterion Validation of the Cross-Cultural Competence Inventory: Final Report. Directed by Dr. Daniel P. McDonald, Patrick AFB, FL: Defense Equal Opportunity Management Institute (DEOMI).

Wersja polska za zgodą autorów: Barzykowski, K., Majda, A., Przyłęcki, P., & Szkup, M. (2019). The Cross-Cultural Competence Inventory: Validity and Psychometric Properties of the Polish Adaptation. PLoS ONE. DOI: 10.1371/journal.pone.0212730

		Zdecydowanie nie zgadzam się				Zdecydowanie zgadzam się
54.	Jestem pewien/a, że potrafię przyzwyczaić się do nietypowych warunków życia w innej kulturze.	1	2	3	4	5	6
55.	Czuję dyskomfort, gdy czyjeś zachowanie lub intencje są dla mnie niejasne.	1	2	3	4	5	6
56.	Dążę do tego, aby dobrze znać swoich sąsiadów.	1	2	3	4	5	6
57.	Nawet kiedy już podejmę decyzję odnośnie czegoś, zawsze jestem chętny/a, żeby rozważyć inną opinię.	1	2	3	4	5	6
58.	Czując stres, potrafię się uspokoić myśląc o innych sprawach.	1	2	3	4	5	6
59.	Przekonania naszego społeczeństwa odnośnie tego, co jest dobre a co złe, mogą nie być odpowiednie dla wszystkich ludzi na świecie.	1	2	3	4	5	6
60.	Kiedy chcę odczuwać mniej negatywnych emocji (smutek, frustracja czy złość) zaczynam myśleć o czymś innym.	1	2	3	4	5	6
61.	Potrafię dobrze współpracować z innymi pomagając im w znalezieniu lepszych sposobów realizacji ich zadań.	1	2	3	4	5	6
62.	Kiedy jem poza domem, lubię chodzić do miejsc, w których byłem/am wcześniej, tak aby wiedzieć, czego mogę się spodziewać.	1	2	3	4	5	6
63.	Ludzie mają różne metody rozwiązywania problemów, które mogą być równie skuteczne.	1	2	3	4	5	6

Dziękujemy za wypełnienie powyższego kwestionariusza i poświęcony czas!

Wersja oryginalna kwestionariusza: Thornson, C. A., & Ross, K. G. (2010, August). The Construct & Criterion Validation of the Cross-Cultural Competence Inventory: Final Report. Directed by Dr. Daniel P. McDonald, Patrick AFB, FL: Defense Equal Opportunity Management Institute (DEOMI).

Wersja polska za zgodą autorów: Barzykowski, K., Majda, A., Przyłęcki, P., & Szkup, M. (2019). The Cross-Cultural Competence Inventory: Validity and Psychometric Properties of the Polish Adaptation. PLoS ONE. DOI: 10.1371/journal.pone.0212730
